# Heterodimers Are an Integral Component of Chemokine Signaling Repertoire

**DOI:** 10.3390/ijms241411639

**Published:** 2023-07-19

**Authors:** Kimia Kaffashi, Didier Dréau, Irina V. Nesmelova

**Affiliations:** 1 Department of Biological Sciences, University of North Carolina, Charlotte, NC 28223, USA; kkaffash@charlotte.edu (K.K.); didier.dreau@charlotte.edu (D.D.); 2 Department of Physics and Optical Sciences, University of North Carolina, Charlotte, NC 28223, USA; 3 School of Data Science, University of North Carolina, Charlotte, NC 28223, USA

**Keywords:** chemokine, chemokine receptor, heterodimer, heteromer, oligomerization, synergy, signaling, binding, glycosaminoglycan, structure

## Abstract

Chemokines are a family of signaling proteins that play a crucial role in cell–cell communication, cell migration, and cell trafficking, particularly leukocytes, under both normal and pathological conditions. The oligomerization state of chemokines influences their biological activity. The heterooligomerization occurs when multiple chemokines spatially and temporally co-localize, and it can significantly affect cellular responses. Recently, obligate heterodimers have emerged as tools to investigate the activities and molecular mechanisms of chemokine heterodimers, providing valuable insights into their functional roles. This review focuses on the latest progress in understanding the roles of chemokine heterodimers and their contribution to the functioning of the chemokine network.

## 1. Introduction

Chemokines play a critical role in regulating cell–cell communications, cell migration, and cell trafficking, not only in normal physiological processes such as development, homeostasis, and immunity, but also in pathological conditions [1,2,3,4,5,6]. Indeed, chemokines contribute to tumor development, including angiogenesis, tumor growth, and organ-specific metastasis [7,8,9,10,11,12]. By forming concentration gradients, chemokine signaling triggers multiple types of cell movement, including migration, haptotaxis, chemokinesis, haptokinesis, and also modulates cell adhesion [1,13,14].

The 48 human chemokines are classified into four subfamilies based on the spacing of the first two conserved cysteine residues: CXC (one amino acid residue, X, apart), CC (adjacent), CX_3_C (three amino acid residues apart), and XC (first of the two cysteines lacking) [1,15,16,17,18]. The nomenclature for chemokines is structured based on their subfamily classification, followed by the letter “L” representing “ligand”, and a subsequent identifying number. Among these subfamilies, CC and CXC are the largest, containing 28 and 17 members, respectively. The two smaller subfamilies consist of only one (CX_3_C) or two (XC) members. In this review, we discuss chemokine heterodimers, referring mainly to CC and CXC chemokines, as current experimental data support only the formation of CC- and CXC-type chemokine heterodimers [19,20,21,22,23,24,25].

Chemokines carry out their functions through interactions with two essential partners: G protein coupled receptors (GPCRs) and glycosaminoglycans (GAGs). GPCRs are characterized by the presence of seven membrane-spanning α-helical segments separated by alternating intracellular and extracellular loop regions [26]. The chemokine receptors belong to class A or rhodopsin-like GPCRs [27] and follow the same nomenclature as chemokines, defined by the chemokine subclass specificity of the receptor [15,28]. For example, human CC and CXC chemokine receptor names consist of the root CCR or CXCR (“R” for receptor), respectively, followed by a number. Following chemokine ligand binding that triggers a conformational change within a receptor, conventional chemokine receptors signal through G proteins and β-arrestins to induce the migration of cells along chemokine gradients [18]. As with other GPCRs, many chemokine–chemokine receptor interactions demonstrate biased signaling: the binding of different ligands to the same receptor leads to the activation of a particular signaling pathway over others, resulting in distinct biological effects [29]. These biased responses can be modulated through changes in the ligand, receptor, and/or the specific cellular context [30]. The conventional chemokine signaling is primarily mediated through the heterotrimeric G protein, especially through Gαi/o, and desensitized by β-arrestin [31,32]. Subsequently, receptors are internalized into endosomes. They are then sorted for recycling or degradation: a process that serves to limit both the magnitude and duration of signaling and facilitate receptor resensitization [31,33,34]. Currently, 19 conventional GPCRs, i.e., signaling via G protein-mediated pathways, are identified in humans. In addition, there are four “atypical” receptors, named ACKR1 through ACKR4, which cannot activate G protein-dependent signaling but utilize β-arrestins to elicit their functions [15,35,36].

The in vivo activities of chemokines are tightly regulated by their interactions with glycosaminoglycans (GAGs), which are highly present on cell surfaces and within the extracellular matrix (reviewed in references [13,37,38,39,40,41,42]). For example, chemokine mutants lacking the ability to bind GAGs were unable to induce cell migration in vivo [39,43,44,45,46,47], and reduced the neutrophil recruitment activity of CXCL8 in the peritoneum, but enhanced this activity in the lung [48]. Among the various types of GAGs expressed in humans, such as heparin/heparan sulfate (HS), chondroitin sulfate (CS), dermatan sulfate, and keratan sulfate, chemokines predominantly bind HS and CS. These interactions between chemokines and GAGs primarily rely on electrostatic forces, where negatively charged GAGs attract chemokines that possess distinct patches of positive charge on their surface [49,50,51,52,53,54,55]. The specific interactions and binding geometry between chemokines and GAGs vary depending on the chemokine, due to differences in the distribution of charged residues [53,54,56]. Importantly, binding to GAGs correlates with chemokine oligomerization, and both play a crucial role in the activity of chemokines [39,57,58,59,60,61,62,63,64,65,66,67,68,69,70].

It has long been recognized that chemokines can form dimers, tetramers, or even higher-order oligomers [60,67,68,71,72,73,74,75]. Initially, the oligomerization of chemokines was considered to have little biological relevance because the dimerization constants typically fell in the micromolar range [20,61,67,71,72,73,76,77,78,79,80,81,82,83,84,85,86,87,88,89,90,91], while average native chemokine concentrations were measured in the nanomolar range [78,92,93,94]. Therefore, chemokine oligomerization was generally attributed to the experimental conditions in biophysical studies that necessitated high protein concentrations [37]. However, it has become increasingly evident that chemokines are heterogeneously distributed in vivo, with local concentrations reaching levels sufficient for dimer and oligomer formation [70,95,96,97,98,99,100,101,102]. For example, CXCL4 and CXCL7 chemokines, which are stored in platelet α-granules, are released in large quantities into the plasma upon platelet activation [98,102]. Moreover, during inflammation, concentrations of certain chemokines increase to direct immune cell trafficking towards the inflammatory sites [93,94,103,104,105,106,107]. As a result, the biological significance of chemokine homodimers and higher-order oligomers has gradually gained recognition [108,109,110,111]. In particular, the experiments demonstrating the activity of CXC-type chemokine dimers [39,48,62,112,113,114,115], the activation of differential pathways by chemokine monomers and dimers [116,117,118], and the stronger affinity of certain chemokine dimers to receptors [119], have challenged the commonly accepted view that monomers are the sole functional biologically relevant species.

Chemokine heterodimers were discovered about 20 years ago [120,121,122], but similarly to homodimers, struggled to gain acceptance. This skepticism likely rose because the functional characterization of chemokines was traditionally based on biological assays using individual chemokines. However, as structurally similar proteins that co-localize spatially and temporally in biological microenvironments, prone to oligomerization chemokines present a unique case. From a “biophysicist” perspective, the selection of a binding partner by a protein from a mixture is naturally driven by energetic favorability rather than the specific protein identity. Consequently, in the presence of multiple chemokines, a dynamic equilibrium is more likely to include not only homodimers and higher-order homooligomers, but also heterodimers and heterooligomers. This review explores the biophysics and biology of chemokine heterodimers and highlights their biological relevance based on recent research reports.

## 2. Biophysical Basis for Chemokine Heterodimerization

### 2.1. Chemokine Monomers and Homooligomers

**Figure 1:**
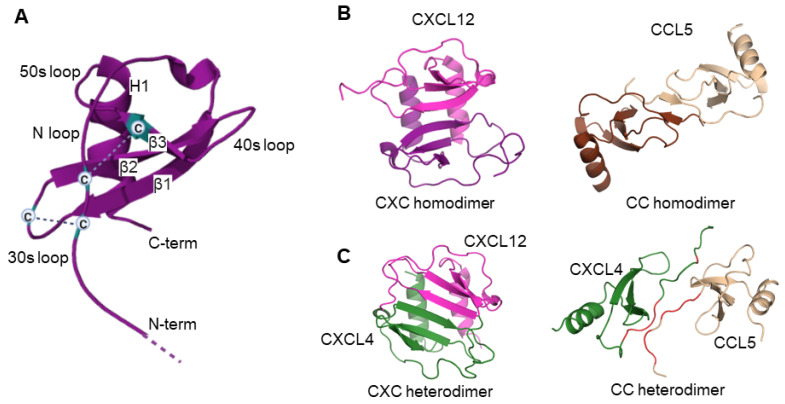
(**A**) Schematic representation of chemokine monomer. Key features are labeled, including N-terminus; N-loop; beta strands β1–β3; alpha-helix H1; 30s, 40s, and 50s loops. Conserved cysteines are shown, and disulfide bonds between them are indicated by dashed lines. (**B**) Structures of CXC (CXCL12, pdb code 2NWG) and CC (CCL5, pdb code 5COY) chemokine homodimers. The monomers are shown in different shades. (**C**) Structural models of CXC (CXCL4-CXCL12) and CC (CXCL4-CCL5) heterodimers. The CXCL4 monomer is shown in green, while the CXCL12 and CCL5 monomers are shown in the same color as in panel B. In CXCL4-CCL5 heterodimer, interface residues are highlighted in red. These residues were determined as those that are located within 4 Å of the opposite monomer.

Despite varying amino acid sequence similarity, which can be as low as 20%, all chemokines adopt a similar fold as monomers [18,90,123,124]. The monomer structure includes an unstructured, flexible N-terminus that precedes the first two cysteine residues; an extended flexible N-terminal loop (N-loop) that follows immediately after them; a three-stranded antiparallel beta-sheet formed by strands β1–β3 and connected by loops referred to as the 30s, 40s, and 50s loops; and a C-terminal alpha-helix (H1) that folds onto the beta-sheet (Figure 1A). Four conserved cysteine residues form two essential disulfide bonds. The first disulfide bond, between the first and third cysteine residues, links the flexible N-terminal part of a chemokine monomer to the 30 s loop (the loop between the β1 and β2 strands). The second disulfide bond, between the second and fourth cysteine residues, links the N-loop to the β3 strand. In addition to linking the N-terminal part and secondary structure elements and holding the chemokine monomer structure together [124,125,126], disulfide bonds play a key role in shaping the characteristics of native state dynamics in chemokines [127,128,129,130].

Structural elements of a chemokine implicated in binding to the receptor were initially identified by the “two-state” model of chemokine–receptor binding that proposed two interaction sites [90,131,132,133]. The first site includes the chemokine N-loop and β3-strand/40s loop that bind to the receptor N-terminus—CRS1 (chemokine receptor site 1). The second site includes the N-terminus of the chemokine that binds to the pocket located within the receptor’s transmembrane region—CRS2 (chemokine receptor site 2). However, subsequent studies have shown that chemokine–receptor interactions may involve additional contact points besides those defined by CRS1 and CRS2, with many of these being specific to individual chemokine ligand–receptor pairing [134]. Moreover, the conformational dynamics of the chemokine’s N-terminal region and the receptor’s binding pocket also affect the chemokine binding to the receptor [112,135,136].

While chemokine monomers are structurally similar, differences between chemokines from different subfamilies become apparent at the quaternary structure level [90,110,124,137]. Members of the CXC subfamily form globular dimers with extensive intermonomer contacts between the first beta-strands of each monomer and the alpha-helices [90,126,137] (Figure 1B, left panel). These dimers are formed by extension of the three-stranded β-sheet from each monomer into a six-stranded β-sheet and the two C-terminal α-helices, running antiparallel folded onto the beta-sheet. On the other hand, CC chemokines form elongated dimers using the N-termini from each monomer, with beta-sheets being positioned across from each other and alpha-helices on the opposite sides of the dimer (Figure 1B, right panel). The CCL20 chemokine is an exception to this general rule as its crystallographic structure exhibits a globular, CXC-type dimer [83,138]. Of note, XC chemokines undergo a rearrangement of the monomeric fold, resulting in four-stranded beta-sheet monomers forming a beta-sandwich dimer [139,140].

Both CXC- and CC-type dimers serve as building blocks for the formation of higher-order oligomers by certain chemokines [60,71,73,75,77], in which chemokines may exhibit both CXC and CC dimer interfaces [60,68,141,142,143]. In addition, higher-order oligomerization enables the diversification of intermonomer interfaces [73,75], leading to the formation of either more globular or more extended quaternary complexes (e.g., CXCL4 or CXCL10 tetramers [73,142] vs. CCL3 or CCL5 [60,68]). Remarkably, even minor variations in the amino acid sequence can give rise to a wide range of oligomeric topologies [60,68,73,144].

The formation of chemokine dimers or higher-order oligomers, and thus the equilibrium of different species, depends on the local chemokine concentrations. While many chemokines tend to dimerize at micromolar concentrations [20,61,67,71,72,73,76,77,78,79,80,81,82,83,84,85,86,87,88,89,90,91], some chemokines remain monomeric even at millimolar concentrations [145,146]. Furthermore, various environmental factors govern chemokine oligomerization, such as pH and ionic strength, as well as the amino acid composition of the intermonomer interface, as demonstrated for several CXC and CC chemokines [72,76,77,81,85,127,138]. The pH-dependence emphasizes the role of electrostatic interactions in chemokine oligomerization. Particularly, the protonation state of the histidine amino acid located at the interface, whose side-chain becomes neutral at basic pH values, frequently influences chemokine dimerization [85,138]. In addition, the protonation of certain carboxylate groups at low pH values can result in electrostatic repulsion [60]. Furthermore, the interaction with sequestering molecules, such as GAGs on the cell surfaces or in the extracellular matrix, plays a crucial role in stabilizing chemokine oligomers [21,39,43,63,64,68,147]. These interactions with GAGs influence the specific type of oligomer that a given chemokine forms [60,148].

### 2.2. Chemokine Heterodimers

Multiple chemokines are abundantly and concomitantly expressed, existing as a dynamic equilibrium of monomeric and oligomeric species regulated by the local environment in normal and pathological microenvironments [1,110,124,149,150]. Experimentally, the heterophilic interactions between different chemokines have been directly detected by several methods, including co-immunoprecipitation and ligand blot [19,24,121,122,151,152,153,154,155], surface plasmon resonance (SPR) [19,121], mass spectrometry [21,122,153], and nuclear magnetic resonance (NMR) spectroscopy [19,20,21,22,23,24,25,156]. The majority of these studies indicated specifically the formation of chemokine heterodimers, although some experimental techniques such as co-immunoprecipitation could only confirm the formation of heteromeric complexes and not differentiate between heterodimers or higher-order heteromers [151,152]. Von Hundelshausen et al. demonstrated the widespread occurrence of heterophilic interactions within the chemokine family. Through a pairwise bidirectional immunoblot chemokine screening, they identified approximately 200 distinct heterophilic interactions and generated a comprehensive map of the chemokine interactome [24].

The structural similarity of chemokine monomers across subfamilies, their tendency to oligomerize, and the structural similarity of homodimers within a subfamily, form the structural basis for chemokine heterodimerization. When the arrangement of amino acid residues at the intermonomer interface becomes more sterically and energetically favorable than in either homodimer, co-localized chemokine homodimers exchange monomers to form a heterodimer [157]. Indeed, the equilibrium heterodimerization constants, determined experimentally for a few chemokine pairs using NMR spectroscopy or SPR, were found to be similar or smaller than the homodimerization constants [19,20,86,121]. Computationally, it has been shown that heterodimerization occurs between chemokines from the same or different subfamilies. In the latter case, the formation of either CC- or CXC-type heterodimer depends on the positioning of specific amino acid residues (positively/negatively charged, polar, or hydrophobic) within the β1 strand and/or the N-terminus [157] (Figure 1C). For example, computational predictions suggested that the CC-type heterodimer of the CXCL4 and CCL5 chemokine pair was more energetically favorable, and the preference towards the CC-type heterodimer was also confirmed experimentally [19,24].

Currently, a complete structural analysis of a native chemokine heterodimer is not available. However, several molecular models of heterodimers have been constructed using experimentally derived constraints. These models rely on the perturbations observed in the NMR chemical shifts of the backbone amide proton and nitrogen in a ^15^N-labeled chemokine upon the addition of an unlabeled heterodimer-forming chemokine partner. The changes in chemical shifts typically occur at or near the intermonomer-binding interface, as they are sensitive to the local chemical environment [158]. Due to the relatively small size of chemokines and their well-defined three-dimensional structure, it is generally assumed that the monomer structure in the heterodimer remains largely unchanged compared to the homodimer. The similarity of chemical shifts in the monomer, homodimer, and heterodimer states strongly supports this assumption [22,23,24,159,160]. Based on this assumption, several molecular models of chemokine heterodimers have been constructed, displaying either the CXC- or CC-type topology of chemokine heterodimers [19,20,21,22,23,24,25,156]. Considering that most chemokine homodimers belong to the CXC- or CC-type structural category, these models are plausible. Nonetheless, given the diverse range of intermonomer interfaces observed in higher-order chemokine oligomers and their sensitivity to even minor residue changes [60,68,73,75,142], the possibility for the deviation of the relative orientation of monomers in certain chemokine heterodimers from that of the homodimers cannot be excluded. This is particularly relevant for CC-type heterodimers, the geometry of which allows for greater flexibility of monomer orientations [144]. Therefore, obtaining an experimental structure of the chemokine heterodimer remains desirable.

## 3. Chemokine Mixtures Trigger Functional Responses Different from Individual Chemokines

Functional studies have revealed that the response from cells elicited by chemokine mixtures can differ from the responses induced by individual chemokines [161]. The ability of chemokine mixtures to exhibit synergistic enhancement or inhibition underscores the complex interplay and unique effects of multiple chemokines in cellular signaling [19,20,24,120,121,162,163,164,165,166,167,168,169,170,171,172,173,174,175,176,177,178]. Several chemokine pairs are highlighted in Table 1 to exemplify the differential response to chemokine mixtures composed of either CXC, CC, or mixed CXC and CC chemokines. Importantly, experiments involving the simultaneous injection of chemokine combinations (CXCL1 and CXCL2 [179], CXCL10 and CCL5 [180]) have shown a synergistic enhancement of leukocyte recruitment in vivo, indicating that synergistic effects are not limited to in vitro settings. These findings underscore the need to go beyond the assessment of individual chemokine functional activities to achieve a comprehensive understanding of the chemokine system.

In numerous instances (Table 1), an altered cellular response to chemokine mixtures correlated with the heterodimerization of chemokines. With regard to CXC chemokines, the formation of CXCL4-CXCL8 heterodimers inhibited CXCL8-dependent signaling in CD34^+^ human hematopoietic progenitor cells [120], enhanced CXCL8-induced migration of CXCR2-transfected Ba/F3 cells [20], and increased the anti-proliferative effect of CXCL4 on endothelial cells [20]. The CXCL12-enhanced chemotaxis of triple negative breast cancer cells (MDA-MB-231) was inhibited in a mixture of CXCL4 and CXCL12 chemokines [24], and CXCL4 counteracted CXCL12-induced ERK (extracellular signal-regulated kinase) phosphorylation in lymphatic and microvascular endothelial cells [185]. Furthermore, CXCL9 and CXCL12, co-expressed in the perivascular tumor, formed heteromers that significantly enhanced the CXCR4-mediated migration of malignant B cells [151]. In the case of CC-type chemokines, the heteromerization with either CCL19 or CCL21 significantly increased the potency of CCL7 chemokine in inducing monocyte migration, with an enhancement of 100-fold or greater [152,166]. Furthermore, CCL19 has been found to enhance CCL22-mediated chemotaxis of human T lymphocytes, with the underlying mechanism most likely being the heterodimerization between the two chemokines [164].

One of the most extensively functionally studied heterodimers, both in vitro and in vivo, is the mixed CXC-CC-type heterodimer formed by CXCL4 and CCL5 chemokines [121]. The heterodimerization of CXCL4-CCL5 has been shown to enhance CCL5-mediated monocyte arrest on endothelial cells, monocyte recruitment to the mouse peritoneum in vivo [121,186], as well as neutrophil recruitment and neutrophil extracellular trap formation [181]. Furthermore, inhibiting the formation of the CXCL4-CCL5 heterodimer impeded the development of atherosclerosis in mice [19,181]. This finding provided the first in vivo evidence of the relevance of chemokine heterodimers and highlighted their potential as targets for the development of novel therapeutics with minimal side effects [187]. Indeed, the inhibition of chemokine heterodimers specifically targets the protein–protein interactions between the chemokine ligands, without interfering with normal ligand-receptor binding and functioning [188]. Following this approach, a CCL5-derived peptide i[VREY]_4_ that mimics the inhibitory effect of another mixed heterodimer, CCL5-CXCL12, was shown to reduce the CXCL12-mediated platelet aggregation in mice [24]. Interestingly, the authors showed that i[VREY]_4_ bound to CXCL12 complexed with CXCR4 and prevented CXCL12-induced Btk (Bruton’s tyrosine kinase) activation, but did not affect pathways required for CXCR4 internalization, consistent with the biased signaling of chemokine receptors [24].

Collectively, the data presented above provide compelling evidence that in environments conducive to chemokine interactions, cellular responses to individual chemokines are likely modified by the presence of chemokine heterodimers or higher-order heteromers. Thus, the inherent propensity of chemokines to oligomerize, as well as readily mix and form heterooligomers in microenvironments where multiple chemokines co-localize, favors the formation of heterodimers alongside homodimers, rather than exclusively homodimer formation. Consequently, a more complete understanding of the roles that chemokine heterodimers play within the chemokine network requires further studies to establish whether chemokine heterodimers act as independent active entities, define their molecular mechanisms, and determine whether these mechanisms resemble or diverge from those driving homodimer activity.

## 4. Obligate Chemokine Heterodimers as Tools to Study Heterodimer Function

In vivo, the concentrations of chemokines can vary significantly both temporally and spatially, resulting in different distributions of monomer, dimer, and heterodimer species regulated by local chemical environments. Therefore, the measured response in in vitro or in vivo experiments reflects the simultaneous contribution from all chemokine species. To assess the unique function of each species, a general approach has been to generate obligate chemokine monomers or homodimers [61,80,113,114,115,116,117,119,147,189,190,191,192,193] and, more recently, heterodimers [23,24,159,160].

### 4.1. Experimental Approaches to Form Obligate Chemokine Heterodimers

Although there is no experimental structure of a native chemokine heterodimer at present, based on symmetry considerations of chemokine homodimers, the currently accepted view is that the heterodimers adopt either the CXC- or CC-type configuration, in which each monomer retains its structural fold. This postulate is supported by the observation of major NMR chemical shift changes at the respective intermonomer interfaces when the heterodimer-forming chemokines are mixed [19,20,21,22,23,24,25] and validated by the stability of CXC- or CC-type heterodimers during MD simulations [23,24,153,157]. Therefore, the straightforward approach to design a non-dissociating, obligate chemokine heterodimer has been to introduce a covalent bond (disulfide [23,159] or oxime [24,160]) at the intermonomer interface so that the structure of the obligate heterodimer is essentially the same as the native heterodimer. Currently, only a few obligate chemokine heterodimers (OHD) are available (Table 2).

**Figure 2:**
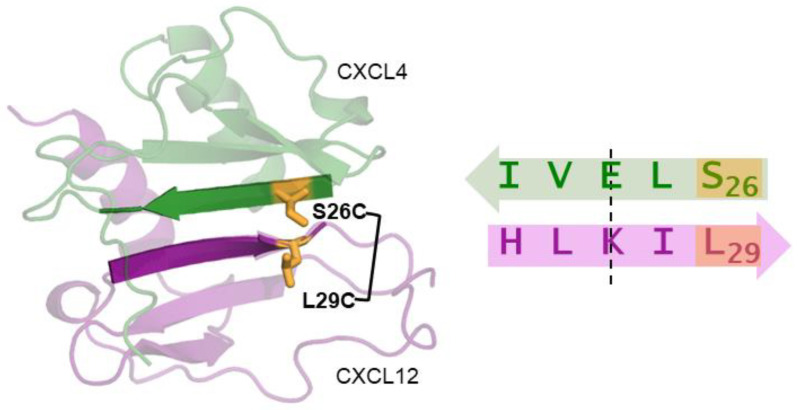
(**Left**) Structural model of the CXCL4–CXCL12 heterodimer. The CXCL4 monomer is shown in green and the CXCL12 monomer is shown in purple. The intermonomer interface formed by the two β1 strands is highlighted, and residues selected for cysteine substitution are labeled and shown in orange with side chains. (**Right**) The amino acid sequence and the direction of the first β1 strand are shown with the axis of symmetry indicated by dashed line. Residues S26 and L29, selected for cysteine substitution, are away from the symmetry axis, preventing the formation of disulfide-linked homodimers.

For the disulfide bond, cysteine amino acid residue substitutions are introduced in each monomer. The strategic placement of these cysteine substitutions away from the symmetry axis prevents the formation of disulfide-linked homodimers. Figure 2 illustrates this disulfide-trapping strategy used to generate the obligate CXCL4-CXCL12 heterodimer, referred to as OHD_4–12_ [159]. The disulfide-trapping approach offers several significant advantages compared to other types of crosslinking methods [194]. In the context of chemokine heterodimers, it allows for isotopic labeling of the heterodimer by expressing and purifying the cysteine mutants of the chemokine monomers from *E. coli*, facilitating subsequent NMR structural studies. Furthermore, unlike bulkier synthetic crosslinkers, the small size of the cysteine side chains minimizes potential conformational heterogeneity or the introduction of artificial conformations to a heterodimer.

The formation of heterodimers through the oxime bond involves incorporating a ketone in one chemokine monomer and an aminooxy functional group in the other [160]. To generate heterodimers such as OPRAH [160] (CXCL4-CCL5) and ORATH [24] (CCL5-CCL17), individual chemokines with ketone or aminooxy modifications are chemically synthesized, and heterodimers are formed using the oxime ligation reaction [160]. The advantages of the oxime ligation method include mild reaction conditions, high chemo-selectivity, and the hydrolytic stability of the oxime bond [195,196]. For instance, in the case of OPRAH, the reaction could be performed with folded proteins in an aqueous buffer, resulting in a yield of 60% [160].

### 4.2. Functional Activity of Obligate Chemokine Heterodimers

Experiments utilizing chemokine mixtures strongly suggest that chemokine heterodimers are active species, and experiments using obligate heterodimers provide direct evidence. In a monocyte arrest assay, OPRAH recruited twice as many monocytes as a mixture of non-covalently associated CXCL4 and CCL5 chemokines [160]. Treatment with the mouse equivalent of OPRAH restored the formation of diet-induced aortic lesions and increased the macrophage content in aortic atherosclerotic plaques in Ccl5^−/−^Cxcl4^−/−^Apoe^−/−^ mice, establishing the in vivo functional activity of chemokine heterodimers [24]. The ORATH chemokine induced T cell arrest during transendothelial migration with higher potency and efficacy than a combination of CCL5 and CCL17 chemokines [24]. Also, while the obligate CXCL4-CXCL12 heterodimer OHD_4–12_ did not demonstrate any significant effect on MDA-MB-231 cell migration on its own, it dose-dependently inhibited CXCL12-driven MDA-MB-231 cell migration [159]. Although experimental evidence remains limited, it is clear that the heterodimers function as active units, and the consequences of their activity depend on the nature of the constituent chemokines, and likely their cognate receptors and the structural and dynamic features of the formed heterodimers.

### 4.3. Limitations of Using Obligate Heterodimers

Obligate heterodimers, similar to obligate homodimers, offer a unique opportunity to assess their function independently of other chemokine species (monomers, homodimers, and homooligomers) that exist in a dynamic equilibrium within chemokine mixtures. However, it is important to consider the limitations associated with the use of obligate heterodimers.

The first limitation pertains to the possible difference, to varying degrees, of the relative orientation of monomers in the obligate heterodimer from that in naturally occurring heterodimers. Indeed, due to the design requirements based on current experimental models, the disulfide bond locks the two monomers in the same CXC- or CC-type geometry as observed in a homodimer. However, following the same logic that leads to the formation of a heterodimer, the distribution of specific residues (e.g., charged and hydrophobic) over the surface of the two monomers may favor a slightly different orientation of the monomers relative to each other than the one that is observed in respective homodimers. This possible variation in the orientation could potentially affect the mode of interaction with the receptor, thereby modifying its response to the heterodimer. Indeed, the use of different crosslinking methods that had a profound effect on the activity of obligate CCL2 dimers was at least partially explained by the deviation from the native dimer structure [190,197,198].

The second limitation is that the formation of a non-dissociating heterodimer restricts the possibility of conformational changes that may occur after binding to the receptor [134], including adjustments in the relative positioning of the two monomers for the optimal fit and receptor activation (receptor binding by the heterodimer discussed in Section 5.1). In fact, this limitation also applies to disulfide-linked homodimers. For example, the CXCR4 N-terminal domain adopts two distinct conformations when bound to the CXCL12 monomer and dimer [199], resulting in different functions for the CXCL12 monomer and dimer [117,199]. The authors proposed that the wrapping of the receptor’s N-terminus around the globular core of the CXCL12 monomer modulated the chemokine’s orientation and interactions with the receptor’s extracellular and transmembrane regions [117,199]. The same principle may apply to obligate chemokine heterodimers.

Consequently, considering these limitations, it would be desirable to allow for additional flexibility in adjusting the monomer orientation, both prior to and following receptor binding.

Nonetheless, despite these limitations, the use of obligate heterodimers is currently the best and least invasive way to dissect activities in chemokine mixtures.

## 5. Molecular Mechanisms of Chemokine Heterodimers

The molecular mechanisms underlying the activity of chemokine heterodimers remain elusive. Potentially, chemokine heterodimers can exert their activity at least by two mechanisms. First, chemokine heterodimers can independently exhibit activity, including the ability to bind and activate chemokine receptors. Second, as a natural part of the equilibrium, heterodimers can modulate the balance between different chemokine species in response to changing in vivo environmental conditions. This, in turn, can have an impact on the overall biological response to the chemokine milieu.

### 5.1. Chemokine Heterodimers Can Bind and Activate Chemokine Receptors

Although chemokine heterodimers have been shown to bind and activate chemokine receptors [20,23,24,154,159], the experimental evidence on heterodimer–receptor interactions is limited, and presently more questions than answers exist regarding these interactions. To firmly establish the stoichiometry of heterodimer–receptor complexes and to determine which pathways can and cannot be activated by the heterodimer, more experimental data are needed. Furthermore, it is important to investigate the relative potency of CXC- and CC-type heterodimers in terms of receptor activation. Indeed, as discussed above, in contrast to CXC-type heterodimers, the inter-monomer interface in CC-type heterodimers involves the N-termini of chemokines (Figure 1C), which limits their availability for the interaction with the receptor [90,190]. Additionally, there is likely a distinction between chemokine receptors in terms of their propensity to bind chemokine heterodimers. For example, CXCL8 chemokine is a ligand for both CXCR1 and CXCR2 receptors [200,201]. The increasing number of CXCL4-CXCL8 heterodimers correlated with the enhanced CXCL8-induced chemotaxis of Ba/F3 cells transfected with the CXCR2 receptor, but not with the CXCR1 receptor [20].

Receptor activation by chemokine heterodimers has been conclusively demonstrated using obligate heterodimers and monitoring downstream signaling events such as the production of cyclic adenosine monophosphate (cAMP) [24] or cytoplasmic calcium (Ca^2+^) release [23,159]. In CCR1-transfected HEK293 cells, the activation of CCR1 receptor by the CXCL4-CCL5 obligate heterodimer OPRAH led to the reduction of cAMP production [24]. The addition of OHD_4–12_ to MDA-MB-231 breast cancer cells induced a dose-dependent increase of cytoplasmic Ca^2+^ with a half-maximal effective concentration (EC50) of 1.3  ±  0.1 nM [159], which was comparable to the EC50 values for wild-type CXCL12 [159,199,202,203]. The addition of the specific CXCR4 inhibitor AMD3100 abrogated the Ca^2+^ release, confirming the activation of downstream signaling of the CXCL12′s receptor CXCR4 by OHD_4–12_ [159]. To test the involvement of CXCR3, the proposed low-affinity CXCL4 receptor [204], the specific CXCR3 inhibitor AMG487, was used. AMG487 had no effect on the Ca^2+^ release induced by OHD_4–12_, demonstrating that OHD_4–12_ did not activate CXCR3 signaling pathways that led to calcium mobilization, at least at concentrations up to 100 nM [159]. However, as the CXCR3 is a low-affinity CXCL4 receptor, its activation at OHD_4–12_ concentrations greater than 100 nM remains to be verified. Alternatively, CXCL4 still may activate other signaling pathways mediated by the CXCR3 receptor. Finally, CXCR4 and CXCR3 receptors can form heterodimers, and whether the OHD_4–12_ or CXCL4-CXCL12 heterodimer formed in situ can bind to the receptor heterodimer, and thereby result in the simultaneous activation of CXCR4 and CXCR3 signaling, also remains unclear. Similar to OHD_4–12_, the chemokine heterodimer CXCL1-CXCL7 induced CXCR2-mediated intracellular Ca^2+^ release [23].

#### Possible Receptor Binding Modes of a Chemokine Heterodimer

Notably, the obligate CXCL1-CXCL7 heterodimer induced the CXCR2-mediated intracellular Ca^2+^ release with potency similar to the individual CXCL7 and CXCL1 chemokines and their mixtures, suggesting that only one monomer of the heterodimer activated the receptor [23]. This observation raises questions about the binding mode of the heterodimer to the receptor(s) of its constituent chemokine monomers, both in terms of geometry and stoichiometry, and the relationship between the binding mode and the altered functional responses in chemokine mixtures.

Biochemical and experimental structural data show that the N-terminus and N-loop of the chemokine interact with the receptor (reviewed in references [133,134,205,206,207,208]). Consequently, CXC- and CC-type heterodimers should exhibit differences in receptor binding. When considering CXC-type heterodimers, it is informative to draw an analogy to the binding of CXC-type homodimers to the receptor, for which experimental data are available [80,115,192,209,210,211,212]. For example, the same residues in both the monomer and homodimer of CXCL1 contribute to the CXCR2 receptor binding, indicating that the residues at the dimer interface are not involved in binding to the receptor [113]. Thus, from the perspective of the CXCL1 monomer, this observation aligns with the proposal that only one monomer in the obligate CXCL1-CXCL7 heterodimer interacts with the receptor [23]. Additionally, the N-terminal part of the CXCR2 receptor interacts with a groove formed by the N-loop and the β3-strand of the CXCL7 monomer, which is located away from the dimer interface [81] (Figure 1A,B). Consequently, from the perspective of the CXCL7 monomer, it can be inferred that the interaction and activation of the receptor by the obligate CXCL1-CXCL7 heterodimer should only require one monomer as well. It is noteworthy that the cryo-EM structure of the CXCR2 receptor complexed with another chemokine ligand, CXCL8, allows the binding of both the CXCL8 monomer and dimer in a nearly identical manner, with minimal involvement of the second monomer and residues at the dimer interface in contact with the receptor [213]. Although the CXCR2 receptor utilized to derive this structure does not include the first 38 amino acid residues, which could potentially affect the positioning of CXCL8, this structure generally supports the notion that a single monomer in the CXCL1-CXCL7 heterodimer is sufficient to activate the CXCR2 receptor.

**Figure 3:**
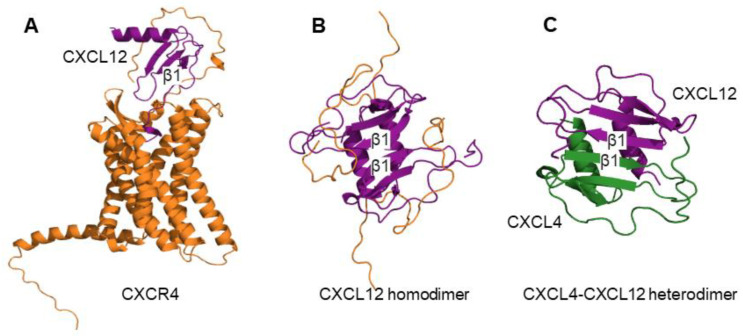
(**A**) Structural model of the CXCL12 chemokine bound to the CXCR4 receptor generated using structures of CXCR4 in complex with a viral chemokine vMIP-II (pdb code 4RWS) and CXCR4 N-terminus in complex with CXCL12 (pdb code 2N55). (**B**) Structure of CXCL12 homodimer in complex with the N-terminus of CXCR4 (pdb code 2K04). (**C**) Structural model of CXCL12-CXCL4 heterodimer. The β1 strands are labeled.

However, the mode of receptor binding appears to vary among different chemokines. Using the obligate monomer (CXCL12_M_) and dimer (CXCL12_D_) of CXCL12, Ziarek et al. [199] demonstrated that CXCL12_M_ and CXCL12_D_ formed separate interfaces with the N-terminal extracellular fragment of the CXCR4 receptor (CXCR4_1-38_, first 38 residues). In the case of CXCL12_M_, the CXCR4_1-38_ wrapped around the monomer, forming a new beta-strand that ran antiparallel to the first beta-strand β1 of CXCL12_M_, thereby extending the beta-sheet (Figure 3A). In contrast, due to the interaction of β1 strands involved in the formation of CXCL12_D_, the CXCR4_1-38_ was unable to make a contact with the β1 strand of CXCL12_D_ [117,199,214] (Figure 3B). It could be hypothesized that similar to the CXCL12_D_–receptor complex, the CXCL4-CXCL12 heterodimer OHD_4–12_, by design, would also prevent the contact of the N-terminus of the CXCR4 receptor and the β1 strand of CXCL12 (Figure 3C). Importantly, CXCL12_M_ and CXCL12_D_ activate distinct signaling pathways mediated by the CXCR4 receptor [117]. While both induce G protein-dependent Ca^2+^ mobilization, cAMP inhibition, and phosphorylation of ERK1/2, CXCL12_D_ only weakly recruits β-arrestin and stimulates the polymerization of the cytoskeletal F-actin [117]. Furthermore, in contrast to CXCL12_M_, CXCL12_D_ inhibits the migration of CXCR4^+^ monocytic leukemia cells [202] and colorectal carcinoma cells [117], and shows an inhibitory effect on the in vivo metastasis of melanoma cells [191]. The observation that the OHD_4–12_ inhibits the migration of MDA-MB-231 breast cancer cells aligns with the inhibitory activity demonstrated by CXCL12_D_, supporting the idea that they may share molecular mechanisms underlying their activity.

In contrast to CXC-type homodimers, the CC-type homodimers cannot bind and activate the receptor because the dimer interface involves N-termini of constituent monomers [190,193]. Yet, OPRAH, an obligate heterodimer of the CC-type, has activated the CCR1 receptor leading to the inhibition of cAMP production [24]. One possibility for OPRAH to retain this activity would be to adjust the conformation upon binding to the receptor to render the N-terminal residues of CCL5 available for the interaction with CCR1. Indeed, the importance of these residues was verified by the lack of activity of OPRAH with N-termini tethered at the first residue [24]. The geometry of the CC-type dimer (Figure 3B,C) is likely more permissive for such conformational adjustments compared to CXC-type dimers because even subtle sequence differences may profoundly affect their quaternary structures [60,68,73,144]. Interestingly, chimeric CC chemokines, which feature a few charged residue substitutions in the β1 strand (not part of the intermonomer interface), acquired the ability to enhance the CCL22-mediated migration of T lymphocytes transfected with the CCR4 receptor [164]. This further suggests that the mutual orientation of monomers within a heterodimer can deviate from classical CXC or CC geometries. In addition, the chemical environment presented by the receptor can promote the change of the relative orientation of the monomers.

Another possibility, which could be especially relevant to chemokine heterodimers formed by chemokine monomers that bind different receptors, would be for a ligand heterodimer to bind to the receptor’s homo- or heterooligomers [161]. Indeed, receptor oligomerization is frequent and associated with altered signaling [187,215,216]. The concept of a ligand heterodimer binding to a receptor heterodimer is not implausible also because the stimulation of the synergistic cell response to certain chemokine ligand pairs correlates with their cognate receptor homo- or heterooligomerization [171,217,218]. Furthermore, forced CXCR4-CCR7 dimerization led to the acquisition of the strong invasive potential by T47D cells in response to CXCL12 and CCL19 in combination [171]. When chemokines CCL5 and CCL17 were combined, the number of CCR4-CCR5 complexes increased compared to the treatment with each chemokine individually [24]. This effect was hindered by a peptide that disrupted CCL5-CCL17 heterodimers and CCR5-derived peptides that mediated CCR5 homodimerization. Peptides disrupting CCL5-CCL17 or CCR5-CCR4 complexes impaired T cell chemotaxis synergy, indicating a potential role of ligand-induced receptor heteromer in this process [24]. Alternatively, as seen for the CCR2-CCR5 receptor heterodimer, receptor heterodimerization may result in negative binding cooperativity, where the binding of one ligand to its receptor inhibits the subsequent interaction of the other ligand [219]. This latter observation also supports the potential relevance of the ligand heterodimer for the interactions with receptor heterodimer.

### 5.2. Interactions with Glycosaminoglycans (GAGs)

Interactions with GAGs likely contribute to the presentation of chemokine heterodimers to chemokine receptors and, by promoting locally increased chemokine concentrations, to their role in modulating the equilibrium between chemokine species. Several studies have indicated that the interactions between chemokines and GAGs promote the formation and stabilization of heterodimers [21,22,23,24,67]. Moreover, the presence of the penta-saccharide heparin-based anti-coagulant fondaparinux has been shown to induce the formation of heterodimers between CCL8 and CCL11 chemokines, which otherwise do not form [21]. The increased formation of chemokine heterodimers in the presence of GAGs aligns with observations for homodimers/homooligomers and, as with homodimers, is likely due to the formation of more extended regions of positive charge upon heterodimerization compared to monomers. However, one can expect that the GAG interactions of heterodimers may differ not only from monomers but also from homodimers. Indeed, the analysis of interactions between disulfide-linked heterodimers CXCL1-CXCL7 or CXCL1-CXCL2 and heparin or heparan sulfate revealed distinct binding characteristics compared to CXCL1, CXCL2, and CXCL7 homodimers [22,23]. For these specific chemokines, a more efficient binding and crosslinking of GAG chains were observed, likely due to the non-equivalent positioning of basic residues on the surfaces of CXCL1, CXCL2, and CXCL7 monomers that led to a more favorable GAG-binding surface.

The biological relevance and link between heterodimer-GAG binding and heterodimer activity are supported by experiments showing that a GAG-binding impaired mutant of CXCL4, which still formed heterodimers with CCL5, did not enhance CCL5-induced monocyte arrest as the wild-type CXCL4 did. Furthermore, the GAG-binding of the CXCL4-CCL5 heterodimers limited the cellular CCR1 receptor internalization, leading to its prolonged availability for G protein signaling [24].

## 6. Summary and Future Directions

This review of recent research demonstrates that chemokine heterodimers are an integral part of the chemokine network and emphasizes the importance of examining chemokine activities within a comprehensive framework that includes chemokine monomers, homo- and heterodimers, as well as higher order homo- and heterooligomers and heterooligomers.

To fully understand how chemokine heterodimers regulate the chemokine system, it is critical to uncover the molecular mechanisms that underlie their mode of action. To that end, research efforts should be directed toward expanding both the type and number of obligate heterodimers available for detailed studies. This would enable the investigation of their biological signaling and facilitate the evaluation of cellular and organismal responses. Moreover, there is a need for structural biology efforts to determine the experimental structure of chemokine heterodimers, establish the binding geometry and stoichiometry of the heterodimer–receptor interaction, and develop molecular model(s) of heterodimer–receptor complexes. Additionally, it is important to decipher the differences within and between CXC and CC subfamilies.

While structural information helps to visualize the mechanistic activity of heterodimers, their functional assessment is crucial for designing effective interventions when the chemokine network becomes dysregulated. Therefore, the identification of context-dependent effects of the dynamic equilibria of chemokine species in various biological normal or pathological conditions is necessary. Currently, the effects of chemokine heterodimers in cardiovascular and tumor environments remain the most studied. The microenvironment, cell type, organ, and the overall health organism status are all likely to play roles in the fine-tuning of signaling responses to chemokine heterodimers. Validating whether specific heterodimers trigger antagonistic or synergistic cell responses also remains an important task. Along with advancing our mechanistic understanding, these functional experiments will aid in the development of therapeutic agents that selectively target chemokine heterodimers. For instance, peptides that mimic the chemokine heterodimer interface hold promise as potential therapeutic agents [19,24,182,183,220,221].

Finally, chemokine interactome may be far more inclusive than initially thought, as new binding partners such as galectins emerge [222,223]. Therefore, a proper interpretation of the biological data requires a more complete understanding of the spatial-temporal variations in chemokine distribution across different microenvironments. Clearly, much more work is needed to understand the mechanism by which chemokine heterodimers form, and how they participate in the chemokine network.

## Figures and Tables

**Table 1:** Examples of chemokine mixtures associated with biological effects on chemotaxis, protein expression or cell phenotype alteration in vitro, and change in in vivo disease models.

Chemokines	Biological Activity Tested	Effect
CXC mixtures		
CXCL1-CXCL2 *	Leukocyte recruitment in rats	enhancement [179]
CXCL4-CXCL8	CXCL8-stimulated HUVEC proliferation	inhibition [20]
CXCL4-CXCL8	CXCR2-transfected Ba/F3 cells chemotaxis	enhancement [20]
CXCL4-CXCL8	CXCR1-transfected Ba/F3 cells or neutrophil chemotaxis	no effect [20,163]
CXCL4-CXCL12	MDA-MB-231 breast cancer cells chemotaxis	inhibition [25]
CXCL7-CXCL8	Neutrophil chemotaxis	no effect [163]
CXCL8-CXCL12	Neutrophil chemotaxis	enhancement [163]
CXCL9/10/11-CXCL12	DLBCL lymphoma cells, pDC chemotaxis	enhancement [151,172,173]
CC mixtures		
CCL2-CCL19	Monocyte chemotaxis	no effect [166]
CCL2-CCL21	Monocyte chemotaxis	no effect [166]
CCL3-CCL5	T lymphoblast chemotaxis in rats	no effect [180]
CCL7-CCL19	Monocyte or CCR7+ dendritic cell chemotaxis	enhancement [166]
CCL7-CCL21	Monocyte or CCR7+ dendritic cell chemotaxis	enhancement [166]
CXC-CC mixtures		
CXCL4-CCL5	Monocyte adhesion on HUVEC	enhancement [121]
CXCL4-CCL5 *	Monocyte and neutrophil recruitment in mice Inhibition of atherosclerosis and aortic aneurysm, preservation of heart function after myocardial infarction, protection against stroke by disrupting CXCL4-CCL5 interaction	enhancement [19,181,182,183]
CXCL6-CCL7 *	Neutrophil recruitment to inflamed tissue in mice	enhancement [184]
CXCL8-CCL2	Neutrophil and monocyte chemotaxis	enhancement [163,165,170]
CXCL8-CCL7	Neutrophil and monocyte chemotaxis	enhancement [163,165,170]
CXCL8-CCL8	Neutrophil and monocyte chemotaxis	enhancement [163,165,170]
CXCL10-CCL3 *	T lymphoblast chemotaxis in rats	no effect [180]
CXCL10-CCL5 *	T lymphoblast chemotaxis in rats	enhancement [180]
CXCL10-CCL22	T lymphocyte chemotaxis	enhancement [164] **
CXCL12-CCL2	Monocyte chemotaxis	enhancement [165]
CXCL12-CCL2 *	IL-10 expression by CCR2+ macrophages, tissue macrophage IL-10 polarization in mice	enhancement [154]
CXCL13-CCL19	CCR7+ or CXCR5+ leukocytes chemotaxis	enhancement [152] **
CXCL13-CCL21	CCR7+ or CXCR5+ leukocytes chemotaxis	enhancement [152] **

Abbreviation: DLBCL—diffuse large B cell lymphoma; HUVEC—human umbilical vein endothelial cells; IL—interleukin; pDC—plasmacytoid dendritic cells. * Studies providing in vivo evidence. ** Paoletti et al. [152] and Sebastiani et al. [164] tested combinations of CCL19 and CCL21 or CCL22, respectively, with 25 different chemokines (not listed).

**Table 2:** Obligate chemokine heterodimers.

Heterodimer	Type	Introduced Covalent Bond
CXCL4-CCL5 [160] (OPRAH)	CXC-CC	oxime
CCL5-CCL17 [24] (ORATH)	CC	oxime
CXCL1-CXCL7 [23]	CXC	disulfide
CXCL1-CXCL2 [22]	CXC	disulfide
CXCL4-CXCL12 [159] (OHD_4–12_)	CXC	disulfide

## Data Availability

Not applicable.
